# Letter to the editor regarding the article “The global distribution of acute unintentional pesticide poisoning: estimations based on a systematic review”

**DOI:** 10.1186/s12889-021-11940-0

**Published:** 2021-10-27

**Authors:** S. Eliza Dunn, Jennifer E. Reed, Christoph Neumann

**Affiliations:** 1 Bayer, Division of Crop Science, 700 Chesterfield Parkway W, Chesterfield, MO 63017 USA; 2 CropLife International AISBL, Avenue Louise 326, Box 35, 1050 Brussels, Belgium

**Keywords:** Pesticide poisoning, Pesticides, Crop protection, Public health, Chemistry, Toxicology

## Abstract

We read with interest the article entitled “The global distribution of acute unintentional pesticide poisoning: estimations based on a systematic review”. We wholeheartedly agree that it is important to evaluate the extent of this issue. We would like to understand the numbers provided in this article, which appear to overestimate the global burden of pesticide poisonings. We also feel that addressing the benefits of these chemistries is important for a complete evaluation.

## Main text

Dear Editor,

We read with interest the article entitled “The global distribution of acute unintentional pesticide poisoning: estimations based on a systematic review” [1]. We wholeheartedly agree that it is important to evaluate the extent of this issue. We would like to understand the numbers provided in this article, which appear to overestimate the global burden of pesticide poisonings. We also feel that addressing the benefits of these chemistries is important for a complete evaluation.

Pesticides are critical for public health. They ensure food security and protect people from insect-borne illness, toxic weeds and carcinogenic mycotoxins which contaminate crops after fungal infections. The World Health Organization (WHO) estimates the global annual burden of malaria and dengue alone to be over 315 million cases [2]. Additionally, the WHO estimates that 690 million people were severely malnourished in 2019 [3]. Pesticides are vital tools for the control of these diseases. Additionally, pesticides are important for farmers to support the social, environmental and economic sustainability needs of society.

In this study, the authors extracted from 157 publications 740,000 annual cases of unintentional acute pesticide poisoning (UAPP) resulting in 7446 fatalities from which they estimated global burden. We are uncertain as to how they arrived at these figures though a substantial proportion of the numbers appear to have been extracted from inflated US data [4–10]. When no data were available for a particular country, the authors extrapolated using UAPP frequencies from other geographies, sometimes relying on sparse, possibly unrepresentative, data. For example, fatal UAPP cases in Western Africa were estimated from data representative of only 0.15% of its population. Similarly, data representing only 2% of the regional population informed the reported non-fatal cases in Central America [1]. The US data seem to have come from American Association of Poison Control Centres (AAPCC) reporting from 2012 to 2017. Although they never establish a case definition of UAPP, it appears the authors have conflated exposure, or coming in contact with a substance, with poisoning, which involves developing adverse symptoms upon exposure. Using the *exposure data* reported by the AAPCC they claim that there are between 72,000–77,000 *poisoning cases* per year. A review of the actual AAPCC exposure data shows degrees of poisoning, ranging from none, to minor, moderate, major and death. From 2012 to 2017 the average annual number of patients exhibiting any poisoning symptom was 15,576 [4–10] -- far fewer than the numbers suggested by Boedeker et al. [1]. Additionally, these numbers combine intentional and unintentional poisonings [4–10], whereas the intent of the paper was to evaluate acute unintentional poisoning. Based on these and the extrapolated data previously mentioned, the authors estimate that 385 million cases of UAPP occur annually world-wide, which we contend overestimates the global burden of UAPP.

Several issues should be addressed in order to establish a more accurate estimation of UAPP. First, the definition of UAPP is inconsistent among included studies. Second, the authors conflate incidence and prevalence values which results in overestimation of annual UAPP cases; cases which occurred prior to the year of interest would be inadvertently included, inflating the final estimate of annual frequency. Third, the authors rely predominantly on self-reported data, which might introduce recall bias to the data. Fourth, as illustrated above, the authors extrapolate potentially unrepresentative estimates to reach national and global counts. This extrapolation has not been validated, nor is it reproducible given the data presented.

This study identifies gaps in knowledge regarding UAPP frequency among geographic regions, and identifies opportunities for the improvement of future studies. However, as discussed above, the study does not establish reproducible numbers nor a validated method for extrapolating the current data. Therefore, the results reported by Boedeker et al. [1] are not strong enough to support policy decisions but have served to point to significant gaps in knowledge. Given this, we would be open to collaborating with the authors on exploring a more robust method for assessment in order to support efforts to reduce the global burden of UAPP.

There is active research to develop viable, less hazardous alternatives to existing pesticides, and ongoing activities to reduce the risk of pesticides in use. Over the past two decades several low-toxicity chemistries have been introduced to the market for crop protection and vector control [11]. Moreover, all new products undergo risk assessments by regulatory agencies before introduction. These assessments include evaluating the conditions of use in low-income markets that comply with the International Code of Conduct on Pesticide Management. A constructive and informed discussion on the role of crop protection and the use of pesticides in sustainable food production is productive and pesticide safety should be addressed in partnership with governments, farmers, NGOs and other stakeholders.

## Data Availability

Not applicable.

## Abbreviations

AAPCC: American Association of Poison Control Centres
NGOs: Non-Governmental Organizations
UAPP: Unintentional Acute Pesticide Poisoning
WHO: World Health Organization
